# The Influence of Prolonged High-Concentration Ozone Exposure on Superhydrophobic Coatings in Static and High-Speed Flow Atmospheres

**DOI:** 10.3390/ma15165725

**Published:** 2022-08-19

**Authors:** Alexander G. Domantovsky, Kirill A. Emelyanenko, Alexandre M. Emelyanenko, Ludmila B. Boinovich

**Affiliations:** A.N. Frumkin Institute of Physical Chemistry and Electrochemistry, Russian Academy of Sciences, Leninsky Prospect 31 Bldg. 4, 119071 Moscow, Russia

**Keywords:** functional materials, ozone, superhydrophobic, oxidation, durability, erosion-corrosion, hydrophobic molecules desorption

## Abstract

The durability of superhydrophobic coatings under exposure to adverse factors that accompany their exploitation in natural and industrial environments remains a key problem in materials science. One such factor is a notable ozone concentration which can be generated as a result of corona discharge, dielectric barrier discharge, piezoelectric direct discharge, UV light photochemical processes, and others characteristic of the power industry. In this work, the mechanisms of degradation of the superhydrophobic coatings under prolonged exposure to high ozone concentrations at dynamic and nearly static conditions were studied. Our results indicate that in dynamic conditions, when the coatings are subjected to joint action of erosion loads initiated by the high-speed flow of the atmospheric air enriched with ozone, with ozone oxidation activity, the superhydrophobic state degrades quite rapidly. At the same time, in nearly static atmospheric conditions with the same ozone content, the degradation is substantially lower. Our study reveals the role of various factors such as the degradation of the layer of the hydrophobic agent, mechanical deterioration of texture, adsorption of ozone, and contact with water in the discovered behavior of the superhydrophobic coatings.

## 1. Introduction

One of the main requirements in the design of new materials with desired properties is to ensure the long-term stability of these properties. Many examples are known where just after manufacturing the material demonstrates high performance; however, under the influence of various destructive factors, the properties of the material continuously deteriorate, which significantly limits the duration and conditions of its reliable operation.

In most cases, the degradation of the functional and structural properties of such materials under the influence of the damaging factors of the environment initiates at the surface of the sample. Therefore, the creation of functional coatings characterized by increased resistance to the destructive effects of an aggressive atmosphere, is a task of current interest. During recent years, the new kinds of passive protective coatings, such as superhydrophobic coatings, are intensively developing in different laboratories throughout the world. In both laboratory experiments and outdoor tests, these coatings have demonstrated numerous functional properties, such as water repellency, resistance to corrosion, anti-icing potential, leakage of current insulating properties, resistance to fouling, etc., [1,2,3,4,5,6,7,8,9,10]. Although a lot of studies estimating the durability of superhydrophobic coatings in different harsh environments have been performed, several issues continue to be unresolved. In particular, the studies of the behavior of superhydrophobic coatings in conditions with high ozone concentration remain scarce [11,12]. Although [12] investigated the interaction of alumina-based superhydrophobic coating with ozone, times and concentrations used were relatively small, while the role of the flow rate was not revealed. At the same time, notable ozone concentration and prolonged exposure in atmospheric and industrial conditions can be resulted from corona discharge, dielectric barrier discharge, piezoelectric direct discharge, UV light photochemical processes, etc. [13]. Such processes are characteristic, for instance, in energy transportation with overhead power lines. The ozone is known as one of the strongest oxidizing agents [14]. Its interaction with metals causes an intense corrosion process, while the interaction of ozone with polymers results in the formation of free radicals, which subsequently leads to the formation of carbonyl, carboxyl, and hydroxyl groups [13,15].

Ozonation-induced corrosion processes can cause undesirable variation in surface morphology and chemistry, while the formation of polar surface groups yields an increase in surface energy. If all above processes are significant, they result in degradation of the superhydrophobic state. That is why it is of great interest and importance for wide industrial applications of the superhydrophobic coatings to study the degree and the mechanisms of degradation of these coatings under the influence of high ozone concentrations. In this manuscript, we present and discuss the evolution of the properties of superhydrophobic coatings on aluminum developed for protection of ambient materials against icing and corona discharge [16] during prolonged exposure in an atmosphere enriched with high ozone concentration.

## 2. Materials and Methods

### 2.1. Materials

The flat coupons of aluminum–magnesium alloy AMg2 (composition in weight %: Mg 2.9, Fe 0.4, Si 0.4, Mn 0.2, Zn 0.2, Cu 0.1, Ti 0.1%, Cr 0.05, total impurities 0.1 and Al to balance, LLC Neva-Meta: Saint Petersburg, Russia) with the sizes of 50 × 50 × 3 mm^3^ were used as substrates for the preparation of the superhydrophobic coatings according to the procedure described in detail in [17]. In brief, for creating the hierarchical surface roughness, the nanosecond laser processing with the laser beam wavelength of 1064 nm was used. The main laser processing parameters were as follows: pulse width 200 ns, pulse frequency 20 kHz, beam velocity 200 mm/s, scanning density 150 lines/mm, single pulse peak power 0.95 mJ in TEM_00_ mode, and focused beam diameter (at the 1/e^2^ level) 40 μm. To decrease the surface energy of laser textured surface, the chemical vapor deposition of the methoxy-{3-[(2,2,3,3,4,4,5,5,6,6,7,7,8,8,8-pentadecafluorooctyl)-oxy]-propyl}-silane at 105 °C was applied inside the sealed cell for 1 h. Further heat treatment of samples resulted in the formation of a crosslinked 2D polymer network of fluorinated molecules chemisorbed onto the surface during fluorooxysilane deposition.

### 2.2. Surface Characterization

To characterize the wettability of our samples and to trace the evolution of a superhydrophobic state during exposure to ozone-enriched atmosphere, we measured the contact and roll-off/sliding angles using a home-fabricated setup (pictures of setups are presented in [18] and Supplementary Materials Figure S1, correspondingly). The applied setup allows accurate determining of the parameters of a sessile droplet and is based on digital processing of the droplet video image, followed by Laplace fit optimization for the droplet shape parameters [18]. Data on the contact angles and roll-off angles for each sample presented in the manuscript were obtained by averaging over corresponding values for 10 droplets deposited at different surface locations. For the contact angle measurements, the 10 μL water droplets were used, whereas for the roll-off angle studies we used the 15 μL water droplets.

To record IR reflectance spectra in the range of 4000–650 cm^−1^, we used a Nicolet 6700 spectrometer (Thermo Scientific: Waltham, MA, USA) and a Smart SAGA (Specular Apertured Grazing Angle) accessory which allows analyzing compounds for IR nontransparent substrates. The angle of incidence was 80°, and the diameter of the circular sampling area was 8 mm. To enhance the spectra sensitivity and accuracy, a mercury cadmium telluride (MCT) detector cooled with liquid N_2_ was used. The spectra were recorded at a resolution of 4 cm^−1^ and obtained by averaging of 256 scans.

The variation in the surface morphology from the sample preparation until the end of experiments on the interaction of the superhydrophobic samples with ozone-enriched atmosphere was traced using a scanning electron microscope (SEM) Nvision 40 (Carl Zeiss: Jena, Germany) at acceleration voltages of 2 kV in secondary electron detection mode. The electron microscope featured an X-Max detector (Oxford Instruments: Abingdon, UK), which was applied to analyze surface chemical composition by the energy-dispersive X-ray spectroscopy (EDS). The EDS spectra were obtained at 5 and 10 kV acceleration voltage.

### 2.3. Ozonation Protocols

The effect of ozonation on the microstructure and properties of coatings was studied for the samples placed for a specified time in the experimental chamber with an air environment enriched with ozone. Two types of conditions were used to mimic possible exploitation conditions. The first type corresponded to exposure of samples to intense flow of ozone-air mixture, when the flow velocity was 10 m/s and the samples were simultaneously subjected to the impact of ozone and the erosion load. The second type of conditions corresponded to the nearly static impact of ozone-enriched atmosphere, when the flow velocity did not exceed 0.1 m/s. Ozone was generated by ozone generator “Ozonbox air-30” (Ozonbox: Chelyabinsk, Russia). The ozone concentration in the air was 0.1 g/m^3^ and remained constant within 10% during the entire series of experiments. During ozonation of the samples, direct measurements of the ozone concentration in the working atmosphere were carried out using an optical gas analyzer F-105 (Optek: Ekaterinburg, Russia). The contact and roll-off angles were used to characterize the degradation of the coating in time during the contact of the samples with static or dynamic ozone-enriched air. The measurements were performed immediately after the sample withdrawal from the experimental chamber with the ozone-enriched atmosphere.

## 3. Results and Discussion

Let us first consider the resistance of the superhydrophobic coatings on aluminum alloy to the degradation in dynamic conditions of interaction with the ozone-enriched atmosphere. As it was mentioned above, the samples were subjected to a high-speed flow of the atmospheric air enriched with ozone, which is a strong oxidizing agent.

**Figure 1:**
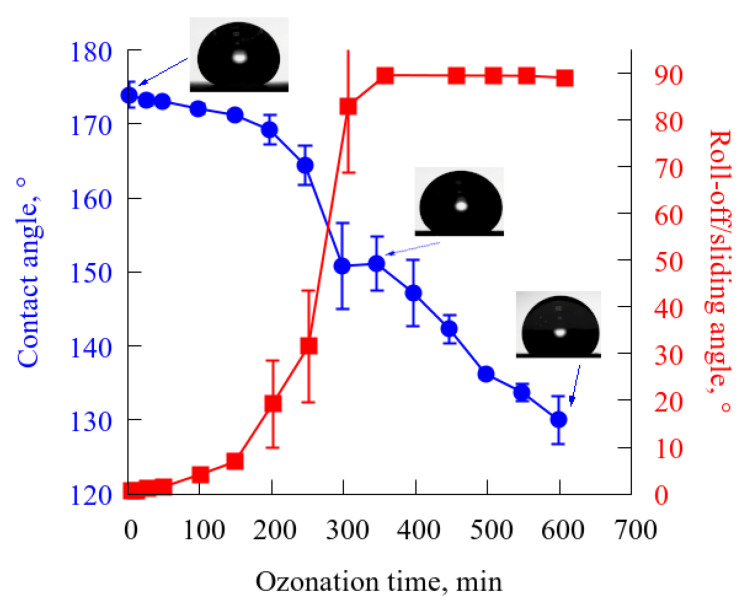
Variation in the contact (blue circles) and roll-off/sliding angles (red squares) with time of exposure to the high-speed flow of ozone-enriched atmosphere.

Data on the variation in the contact and roll-off/sliding angles with time of exposure to the flow of ozone-enriched air presented in Figure 1 indicate relatively rapid degradation of the superhydrophobic state of the samples. Thus, after 300 min of ozonation, the droplet rolling along the surface was replaced by the droplet sliding indicating the partial loss of heterogeneous regime of wetting, while after 350 min of ozonation, the contact angle dropped below 150°. It is interesting to note that at this stage of experiment, termination of the impact of the ozone flux and keeping the sample under atmospheric conditions during 90 min allowed the sample to self-repair with the contact angle of 165.1 ± 0.7 and the roll-off angle of 22.3 ± 3. Thus, short-term self-repairing allowed obtaining the wettability characteristics close to the initial values. However, more prolonged than 350 min ozonation caused further degradation, accompanied by the transition from the heterogeneous to homogeneous wetting regime (Figure 1). After 600 min of ozonation, the contact angle dropped to 129.8 ± 3.3°. Although further 90 min of self-repairing resulted in contact angle increase to 143 ± 0.7°, the homogeneous wetting regime preserved with water droplets pinning to the surface even at 90° of surface inclination to horizon.

The observed degradation of the superhydrophobic coatings in above experiment can be related to the joint action of two different factors. Namely, high-speed flux of an atmospheric air, which is typically enriched with highly abrasive dust particles causes notable erosion of surfaces [19,20,21].

Additionally, high oxidizing ability of the ozone causes both the corrosion of the underlying metal substrate and the oxidation with further desorption of the hydrophobic molecules from the surface, followed by its hydrophilization. Thus, in the above-described experiment, there is mechanical wear by solid particles in combination with electrochemical processes through the action of corrosive ozone medium, with both actions happening concurrently in a dynamic flow system [20]. On the other hand, the increase in the surface energy due to the hydrophobic agent desorption and removal takes place. The conditions used in this experiment were extremely adverse because the ozone flux had very high speed and thus the abrasive particles had high kinetic energy leading to the erosion of the material. Moreover, the ozone concentration was more than two orders of magnitude higher than the typical atmospheric ozone concentration [16,22]. For example, the highest summer ozone concentration in Moscow in the near-surface atmosphere reached 259 μg/m^3^ in 2002 [23]. To differentiate the impact of erosion-corrosion and the oxidizing activity of the ozone-enriched atmosphere, we performed the experiment according to the second protocol. As discussed in the Section 2.3. “Ozonation protocols”, the flow rate for this protocol was less than 0.1 m/s, which allowed avoiding the erosion degradation in these experiments.

**Figure 2:**
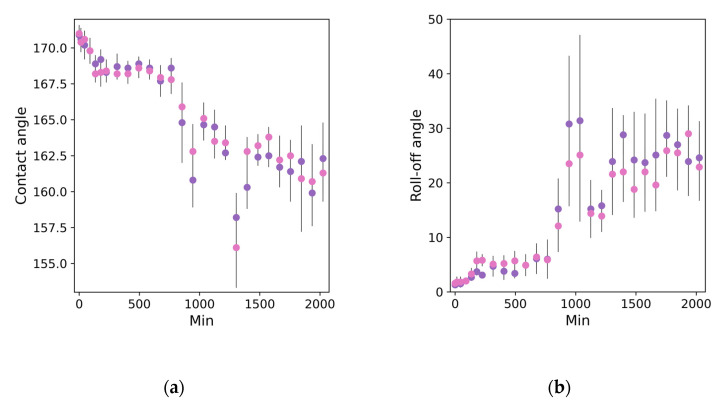
The impact of ozonation time on the values of contact (**a**) and roll-off angles (**b**) of tested samples. Different colors correspond to two different samples tested simultaneously.

The variation in the contact and roll-off angles upon ozonation in this case are presented in Figure 2. The obtained data unambiguously indicate significantly slower degradation of the superhydrophobic state in the stationary atmosphere with a very high-concentration of ozone than in the experiments with high ozone flow speed. During 15 h of ozonation, the evolution of the wettability was low with contact angles remaining higher than 165° and roll-off angles less than 7°. However, further continuation of ozonation leads to a change in the kinetics of degradation, characterized by continuous deterioration of the contact angles. As for the roll-off angles, they showed the transition to sliding angles with rapidly increasing the values to 90° (not shown in Figure 2b). At the same time, it was found that 10 min washing of samples with water just after ozonation lead to the return of droplets rolling along the surface with the preservation of values of the roll-off angles less than 30° (Figure 2b). Please note that in Figure 2b for ozonation times higher than 1000 min, we presented the roll-off values measured after samples washing in water and air drying for 10 min.

Thus, the comparison of the degradation rates detected using two different measurement protocols allows concluding that the erosion-corrosion taking place in high-speed flows of ozone is more harmful for the superhydrophobic state than the oxidation activity of ozone itself. Now let us consider the processes taking place during the interaction of the superhydrophobic samples with the nearly stationary atmosphere saturated with ozone. To estimate the changes in the very top layer of our tested samples, the grazing reflection IR spectroscopy was used.

**Figure 3:**
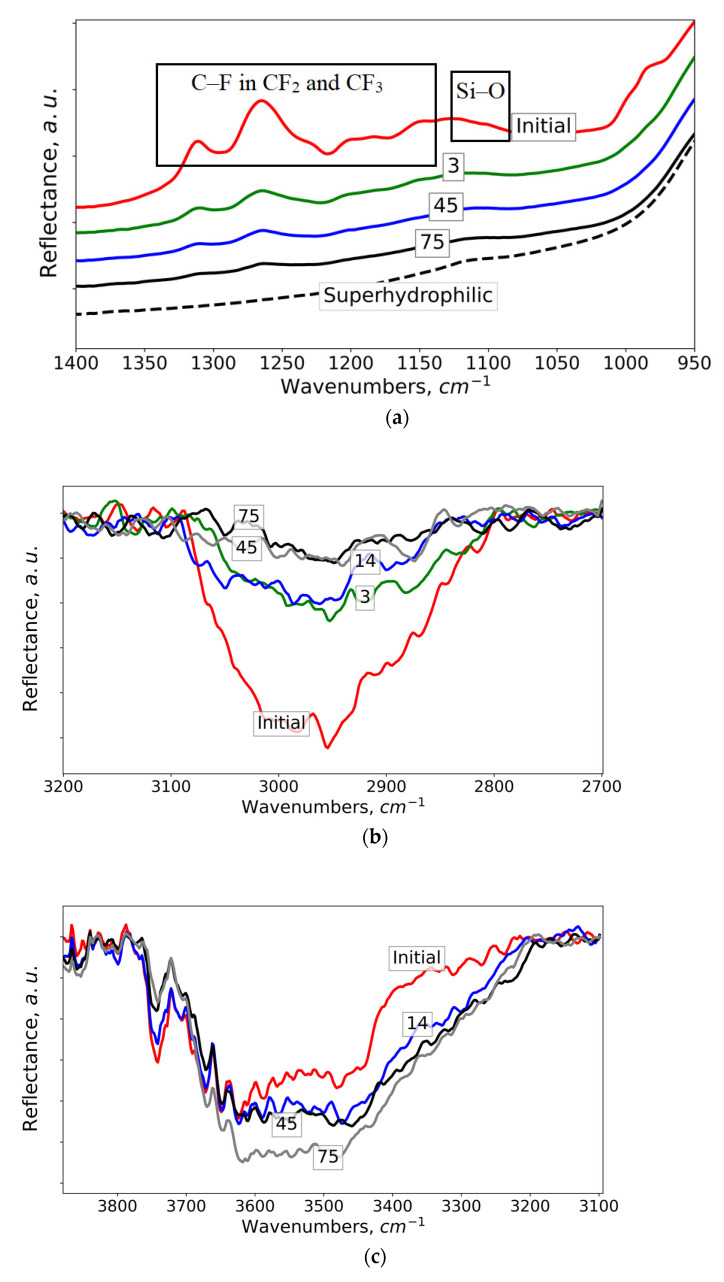
The evolution of the vibration bands intensity with the increase in the time of ozonation. (**a**) Range of C–F and Si–O stretching vibrations; (**b**) range of C–H stretching vibrations in CH_2_ groups; (**c**) range of stretching O–H vibrations in adsorbed water molecules. The numbers on lines indicate the total time of ozonation (in hours) before the spectra measurement.

Spectroscopic data presented in Figure 3a,b indicate the decrease in the quantity of the methoxy-{3-[(2,2,3,3,4,4,5,5,6,6,7,7,8,8,8-pentadecafluorooctyl)-oxy]-propyl}-silane on top of textured elements and inside the nanopores. Note, that as it was detected in [2,17], in the course of the superhydrophobic samples preparation, the fluorooxysilane chemisorbed onto the surface of texture elements from the vapors and condensed with further encapsulation into nanopores. The interaction of the fluorooxysilane with ozone leads to the partial breaking of O-Si bonds linking the hydrophobic molecules to the surface and the desorption of these molecules from the surface. The conclusion that the break of the chemical bond occurs precisely along the O-Si bond is based on the fact that we observed a gradual decrease in the heights of all bands associated with oscillations in the hydrophobic tail of the fluorooxysilane molecules. To demonstrate this process, in Figure 3a the evolution of the vibration bands intensity with the increase in the time of ozonation is shown for C-F, Si-C and Si-O vibrations in CF_2_, CF_3_, Si-CH_2_, and Si-O-Si groups [24,25]. For the comparison, the spectrum of hydrophilic sample (before deposition of the hydrophobic agent) is shown in Figure 3a by a dashed line. In Figure 3b the range of C-H stretching vibrations in CH_2_ groups is presented. The numbers on the spectra indicate the total time of ozonation before the spectra measurement. At the same time, the fact that the coatings preserve high contact angle (>160°) unambiguously indicates that even after 75 h of exposure to ozone, the hydrophobic molecules are present on the surface in a sufficient amount. The analysis of the range of spectra related to O-H stretching vibrations (Figure 3c) reveals some minor increase in the intensity of bands associated with IR activity of monomer and dimer adsorbed water molecules, indicating the adsorption of individual water molecules onto the superhydrophobic surface in contact with the ozone-enriched atmosphere for a long time.

Another mechanism contributing to the contact angle decrease and corresponding increase in the roll-off angles can be related to the adsorption of ozone onto the textured surface and its accumulation inside the nanocavities of the relief. This hypothesis is substantiated by the following experimental findings: It was found that the samples subjected to prolonged ozone exposure (>1000 min) released ozone, which can be detected by the specific odor of the sample characteristic of ozone. The adsorption and encapsulation of ozone by a rough aluminum substrate with micro- and nanotexture was shown in our earlier study [12] by means of FTIR spectroscopy. That study demonstrated high accumulation of ozone by superhydrophilic substrates and much less accumulation by a superhydrophobic aluminum alloy surface. Moreover, it was found that only prolonged exposure to ozone-enriched atmosphere causes the detectable adsorption of ozone. Such ozone molecules adsorbed on the surface or incapsulated by nanopores are easily hydrated with water leading to a decrease in the contact angle and the transition from the “lotus effect” to the “rose petal effect”. At the same time, ozone is known to be highly soluble in water; this ozone property is associated with the observation that washing by water of the sample which was in prolonged contact with the ozone-enriched atmosphere leads to odor disappearance and transition from water droplets pinning to the surface or large sliding angle to roll-off angles less than 30°.

**Figure 4:**
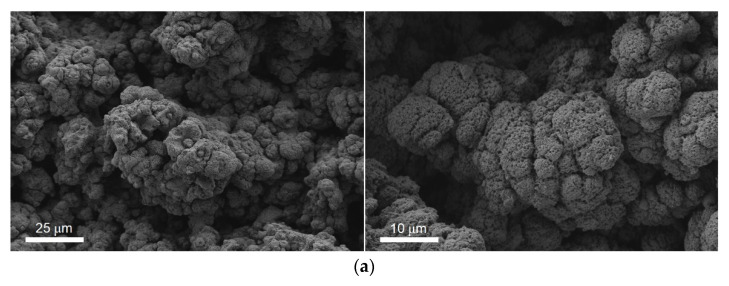

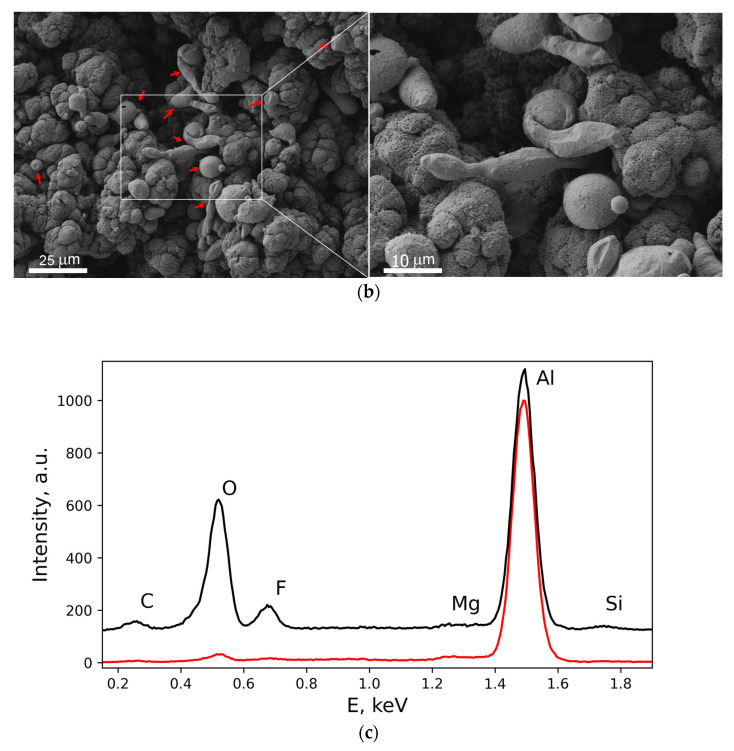
(**a**) SEM images of as-prepared superhydrophobic coating. (**b**) Wettability defects (shown by red arrows) on top of superhydrophobic coatings found after 75 h of continuous exposure to the atmosphere enriched by high ozone concentration. (**c**) EDS spectra registered from the area with porous layer (black line) and from patches without a porous layer (red line).

To learn more about the evolution of the state of the surface of superhydrophobic samples, we studied the variation in the morphology of the surface textures during its contact with ozone. It was found that during ozonation the defective regions appeared on the sample surface which were associated with microtexture elements not decorated by a porous layer (Figure 4b). This porous layer was initially formed by nanoparticles deposited from the laser plume onto the heated surface in the process of laser ablation of the material (Figure 4a). According to the results of elemental and phase analysis [17], the composition of this layer basically corresponds to the formula Al_2_O_3_ with aluminum oxynitride nanoinclusions.

It is worth noting that for the industrial grade materials, various kinds of defects and inclusions are almost always present in the bulk and on the surface. Despite the fact that the process of laser surface treatment is highly automated and well-reproducible, defective patches may appear. In such patches, the conditions for the optimal formation of the upper porous layer can be violated which leads to a weakening of the adhesion of the porous layer to the underlying substrate. In the process of ozonation, the high oxidative activity of ozone contributes to the corrosion-induced decrease in the adhesion of the nanotextured layer to the microtextured surface for such defect patches. The spontaneous removal of this layer results in exposing smooth patches the composition of which is close to that of the original material.

Patches without a porous layer (shown by red arrows) are hydrophilic. The absence of the hydrophobic molecules in such patches were confirmed by the EDS analysis.

As a result, the surface of the sample becomes inhomogeneous, since small patches with hydrophilic properties appear on the superhydrophobic surface. The images presented in Figure 4b clearly show that the linear size of such patches can reach tens of micrometers. The hydrophilic region serves as an effective center for water droplets pinning, which also contributes to an increase in the roll-off/sliding angle for the samples ozonated during the prolonged time and transition from “lotus effect” to the “rose petal effect”.

## 4. Conclusions

In this study, we aimed to analyze the degree and the mechanisms of degradation of the superhydrophobic coatings under the influence of high ozone concentrations. We used the superhydrophobic coating which was fabricated based on pulse laser processing and already demonstrated high resistance to many kinds of destructive loads [16,17]. We intentionally ran experiments at very high ozone concentrations to simulate the behavior of our coatings under extreme conditions which may occur during operation, both in natural and industrial conditions, only as a short-term phenomenon. Our results indicate that in dynamic conditions, when the coatings are subjected to joint action of erosion loads initiated by high-speed flow of the atmospheric air enriched with ozone, with ozone oxidation activity, the superhydrophobic state degrades quite rapidly. In this case, the transition from the superhydrophobic state to the highly hydrophobic one already occurs after 5 h of exposure.

The comparison of the degradation rate of the superhydrophobic coatings during ozone exposure in dynamic and nearly static conditions allowed us to associate a rapid degradation process in dynamic conditions to a strong impact of the erosion-corrosion. As for the evolution of the properties of superhydrophobic coatings in a nearly static atmosphere enriched with high ozone concentration, it is rather weak during first 16 h of exposure. Further continuous impact of ozone causes some notable degradation, mainly revealed by an increase in the roll-off angles and the transition to a partial homogeneous wetting regime. At the same time, it was found that these processes can be suppressed by simple water washing of samples. The latter indicates a self-repairing function under raining for the superhydrophobic coatings exploited in atmospheric conditions. Thorough IR spectroscopy and SEM analysis allowed explaining the observed degradation of the superhydrophobic coatings in the process of stationary ozonation by two factors. These are the partial oxidation and desorption of fluorooxisilane from the texture, and the corrosion-induced decrease in the adhesion of the nanotextured layer to the microtextured surface leading to its spontaneous removal from separate patches of the surface. To summarize the chemical effect of ozone on the evolution of the superhydrophobic state of aluminum alloy surface, a schematic diagram is provided in the Supplementary Materials in Figure S2.

However, it is worth noting that the above processes are dependent on ozone concentration and thus, in ordinary exploitation conditions, will hardly result in notable degradation of the superhydrohobic state.

## Data Availability

Not applicable.

## Supplementary Materials

The following supporting information can be downloaded at: https://www.mdpi.com/article/10.3390/ma15165725/s1, Figure S1: Setup for measuring roll-off/sliding angles; Figure S2: Schematic diagram of mechanism of the O_3_-induced superhydrophobicity deterioration.
