# Supplementary material for: The Influence of Prolonged High-Concentration Ozone Exposure on Superhydrophobic Coatings in Static and High-Speed Flow Atmospheres
Source: Materials (Basel). 2022 Aug 19;15(16):5725. doi: 10.3390/ma15165725 (PMC9416390; doi:10.3390/ma15165725)
Supplement: Supplementary file 1 [file materials-15-05725-s001.zip › materials-1861535-supplementary.pdf]

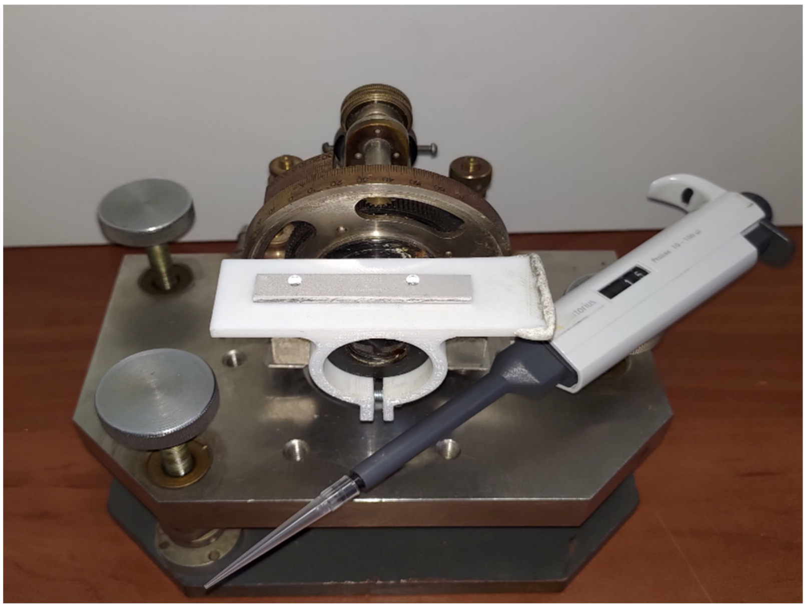

Figure S1. Setup for measuring roll-off/sliding angles.

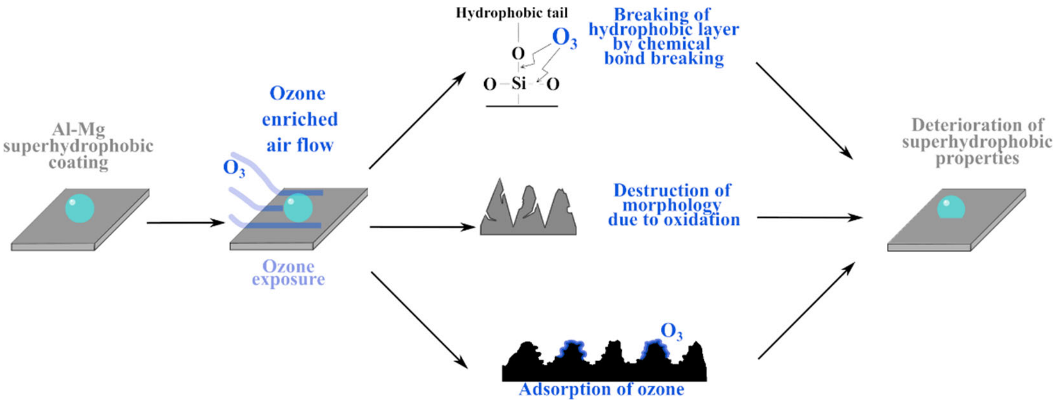

Figure S2. Schematic diagram of mechanism of the O_3-induced superhydrophobicity deterioration.
